# Long-term prognostic value of native myocardial tissue relaxation parameters (T1, T2, and T1ρ) in patients with precapillary pulmonary hypertension

**DOI:** 10.1007/s10554-025-03451-5

**Published:** 2025-07-05

**Authors:** Yue Wang, Ying Zhang, Srikant K. Iyer, Walter RT Witschey, Yuchi Han

**Affiliations:** 1 Cardiovascular Division, Department of Medicine, Perelman School of Medicine of the University of Pennsylvania, Philadelphia, PA, USA; 2 Department of Cardiology, Shanghai Ninth People’s Hospital, Shanghai Jiaotong University School of Medicine, Shanghai, China; 3 Department of Cardiology, PLA General Hospital, Beijing, China; 4 Department of Radiology, Perelman School of Medicine of the University of Pennsylvania, Philadelphia, PA, USA; 5 Cardiovascular Division, Department of Internal Medicine, The Ohio State University Wexner Medical Center, Columbus, OH, USA; 6 The Ohio State University Wexner Medical Center, 460 West 10th Ave, Columbus, OH 43210, USA

**Keywords:** Pulmonary hypertension, Tissue characterization, CMR, Mapping, T1, T2, T1ρ

## Abstract

Quantitative native cardiovascular magnetic resonance imaging (CMR) techniques such as T1, T2, and T1ρ mapping can provide myocardial tissue characteristics non-invasively. The objective of this study was to evaluate the alterations of T1, T2, and T1ρ in patients with precapillary pulmonary hypertension (PH) and their prognostic value. We prospectively enrolled 35 PH patients and 15 healthy controls between 2014 and 2017. PH patients were followed for all-cause death. CMR functional and tissue relaxation parameters were acquired and analyzed. Multivariate Cox regression models and time-dependent receiver operator characteristic (ROC) curves were constructed to determine the risk assessment/predictive value of T1, T2, and T1ρ for all-cause mortality. During a median follow-up of 79 months (Interquartile range 40–103 months), 15 out of 34 patients died. Compared with healthy controls, T1, T2, and T1ρ values were significantly higher in patients with precapillary PH. T1, T2, and T1ρ values at right ventricular (RV) insertion points (IPs) showed the high correlation with RV functional parameters. T1_RVIP showed the highest predictive value in time-dependent ROC analysis for all-cause mortality during the entire observation time (3–9 years). T2_RVIP of 56.1 ms showed the highest value predictive of 3-year mortality with an AUC of 0.950 (sensitivity: 1.000, specificity 0.824) and T1_RVIP of 1215.2 ms had excellent discrimination of AUC of 0.870 (sensitivity: 0.946, specificity 0.922). According to Kaplan-Meier analyses, a native T1_RVIP of 1215.2 ms (*P* = 0.003), a native T2_RVIP of 56.1 ms (*P* = 0.002), and a native T1ρ_RVIP of 109.5 ms (*P* = 0.005), were predictive of all-cause mortality. In univariate Cox regression models, T1_RVIP, T2_RVIP, and T1ρ_RVIP, as well as RVEDVi and NT-proBNP, are predictive of all-cause-mortality. In multivariate analyses, T2_RVIP was the sole independent predictor for mortality (hazard ratio, 2.5, [1.1–5.9], *P* = 0.035). Native tissue relaxation parameters T1, T2, and T1ρ at RVIPs are highly correlated with RV functional parameters and may have prognostic value. T2_RVIP is independently predictive of all-cause mortality in precapillary PH during long-term follow-up in this small prospective study.

## Introduction

Pulmonary hypertension (PH) is a progressive life-threatening disease which affects 1% of the global population [1]. During the past 20 years, the mortality rate of PH has increased by 2% annually [2]. The 3-year mortality rate of the high-risk group is up to 28–55% [3]. Precapillary PH is defined as having a mean pulmonary arterial pressure > 20 mm Hg, pulmonary arterial wedge pressure ≤ 15 mm Hg, and pulmonary vascular resistance (PVR) ≥ 3 Wood units by right heart catheterization, which includes all pulmonary hypertension groups except group 2, which is caused by left heart disease [4, 5]. Although the exact etiological mechanisms are different, precapillary PH involves vascular remodeling and increased pulmonary vascular resistance results in sustained right ventricular overload, leading to RV enlargement and dysfunction, and eventually, RV failure and death. Decreased RV function is the most powerful underlying determinant of morbidity and mortality in patients with precapillary PH [6, 7].

Cumulating evidence suggests that intrinsic myocardial impairment and remodeling, triggered by RV pressure-overload, are fundamental mechanisms and occur before RV dysfunction that could be detected clinically [8]. Inflammatory infiltration, cardiomyocyte cell loss, myofibroblast activation, extracellular matrix remodeling, and diffuse fibrosis have been observed by tissue analyses of endomyocardial biopsies, explanted hearts, or autopsy material [9-11].

Cardiovascular magnetic resonance imaging (CMR) is the leading imaging modality for cardiac structure and function, especially myocardial tissue characterization. The presence of late gadolinium enhancement (LGE) at RVIP has been shown to be a predictor of time to clinical worsening in patients with pulmonary hypertension, which highlight a close relationship between cardiac tissue characteristics and cardiac function and outcomes [12]. Unlike LGE, native tissue mapping is more sensitive to diffuse fibrosis and myocardial histological changes. Quantitative parameters of T1, T2, and T1ρ values offer opportunities for describing intrinsic myocardial tissue properties at any regions of interest, without exogenous contrast agents. Elevated T1 values were observed at RVIP before LGE is detectable and before the deterioration of RV function [13]. We and others have reported increased T1_RVIP and T2_RVIP and associated with function and hemodynamic parameters in PH patients [14-16]. Recently, increased T1ρ relaxation time have been reported by us and others and correlated with LGE in HCM patients [17, 18]. However, it is still unclear whether these tissue characteristics are independently prognostic in precapillary PH patients. In this study, we aimed to assess the correlation of native T1, T2, and T1ρ with ventricular functional parameters, and the relationship of these parameters with all-cause mortality in precapillary PH patients.

## Methods

### Patients and follow-up

We prospectively enrolled patients with precapillary PH to undergo CMR, including function imaging and tissue characteristic imaging (T1, T2, and T1ρ) between January 2014 and February 2017. These patients were identified according to 2013 World Health Organization (WHO) criteria based a prior right heart catheterization with mean pulmonary arterial pressure ≥ 25mmHg, a pulmonary capillary wedge pressure ≤ 15 mmHg, and pulmonary vascular resistance ≥ 3Wood unit [5]. In addition, they were of WHO functional class II, III, or IV, and on stable pulmonary arterial hypertension-specific medications for 3 months without dose changes within 28 days of CMR. Patients were excluded if they had CMR contraindications, receiving intravenous inotropes within 2 weeks of baseline imaging, severe lung diseases, or portal hypertension with severe liver disease. The 15 controls were healthy volunteers without any history of hypertension, hyperlipidemia, diabetes, or any other cardiovascular or pulmonary diseases and were on no cardiovascular medications. The end point was all-cause mortality and patients were followed until August 2023. This study was approved by our Institutional Review Board and all subjects gave written informed consent.

### CMR protocol

CMR was performed on a single 1.5 T MRI scanner (Avanto; Siemens, Germany) equipped with 32-channel anterior and posterior receiver arrays. Contiguous cine short-axis slices were acquired using steady-state free precession imaging (SSFP) to assess functional parameters in both ventricles. Retrospective ECG-gated cine imaging was performed with the following parameters: TE/TR = 1.07/2.2 ms; slice thickness = 8 mm; slice gap = 2 mm; bandwidth = 930 Hz/pixel; flip angle = 70°; field-of-view = 320–380 mm^2^; spatial resolution = 1.8–2.0 mm^2^; parallel imaging factor = 2.

Native T1 maps were acquired at mid-ventricle using a modified Look-Locker inversion recovery (MOLLI) sequence with the 5(3)3 scheme [Siemens works-in-progress 448] using the following sequence parameters: TE/TR = 1.17/2.4 ms; minimum TI = 100 ms; TI increment = 80 ms; flip angle = 35°, field of view = 320–380 mm^2^; spatial resolution = 1.5–2.0 × 1.5–2.0 mm^2^; slice thickness = 8 mm; bandwidth = 1,085 Hz/pixel; parallel imaging factor = 2.

T2 maps were acquired at the mid-ventricle using a T2- prepared SSFP sequence with the following sequence parameters at end-systole: T2-preparation durations = 0, 24, and 55 ms; TE/TR = 1.37/2.64 ms; flip angle = 35°; field of view = 320–380 mm^2^; bandwidth = 1,185 Hz/pixel, matrix = 192 × 120 pixels, spatial resolution = 1.9–2.3 mm^2^ slice thickness = 8 mm; parallel imaging factor = 2.

T1ρ maps were acquired at the mid-ventricle using eight T1ρ weighted images with different spin locking pulse duration (TSL) in a single breath hold: TSL = 0, 2, 10, 18, 26, 34, 42, 50 ms, B1 = 400–500 Hz, spatial resolution = 1.4 × 1.4 mm^2^ slice thickness = 8 mm, flip angle = 70°, TE = 1.45 ms, TR = 2.9 ms, bandwidth = 900 Hz/pixel, linear k-space phase encoding ordering, parallel imaging with acceleration factor = 2, two heartbeats for T1 relaxation between shots [17].

### Image analysis

LV and RV endocardial and epicardial border were manually traced on short axis slices according to SCMR recommendations using Qmass software (Medis, Netherlands) [19]. Papillary muscles were included in the ventricular volume. RV mass measurements were performed during end systole as described [20]. For T1, T2, and T1ρ analysis, regions of interest (ROIs) were manually drawn at upper RVIP, lower RVIP, septum (mid septum between the insertion points), LV lateral wall, and RV free wall (Aquarius iNtuition, TeraRecon, Foster City, USA) (Supplemental Figure S1). Average RVIP was calculated as: (upper RVIP values + lower RVIP)/2.

### Intra- and Inter-observer reliability

Ten patients were randomly selected to assess for intraobserver variability for both the CMR functional measurement and mapping parameters. The observer was blinded to the initial measurements and conducted the repeat measurements 4 weeks later. For inter-observer reliability, the same 10 patients were analyzed by another reader blinded to the results of the first reader.

### Statistical analysis

Continuous variables are shown as mean ± standard deviation (SD). Categorical variables are presented as percentages. The differences between PH patients and controls were calculated by Wilcoxon rank-sum test. Spearman’s correlation was used to assess the linear correlation between tissue mapping parameters and functional parameters. The reliability of the different approaches was assessed by intra class correlation coefficient (ICC) analysis. The cutoff values of T1, T2, and T1ρ at RVIP was determined using Youden’s index with receiver operating characteristic (ROC) analysis. Kaplan-Meier curve was used to evaluate patient outcome using the cutoff values of T1, T2, and T1ρ values at RVIP. Univariate Cox regression was used to calculate hazards ratios (HRs) for outcomes. Multivariate Cox proportional hazards models were performed to determine independent associations with the outcomes. Due to the small sample size and events in this study, two variates (one functional parameter with the lowest p-value and one mapping parameter) were included into multivariate Cox regression analysis in addition to age and sex to ensure the stability of the model. A p-value of < 0.05 was considered statistically significant. Data analysis was performed using the SPSS 17.0 (Inc, Chicago, USA) and GraphPad Prism 5 (La Jolla, CA).

## Results

### Patient characteristics

**Fig. 1:**
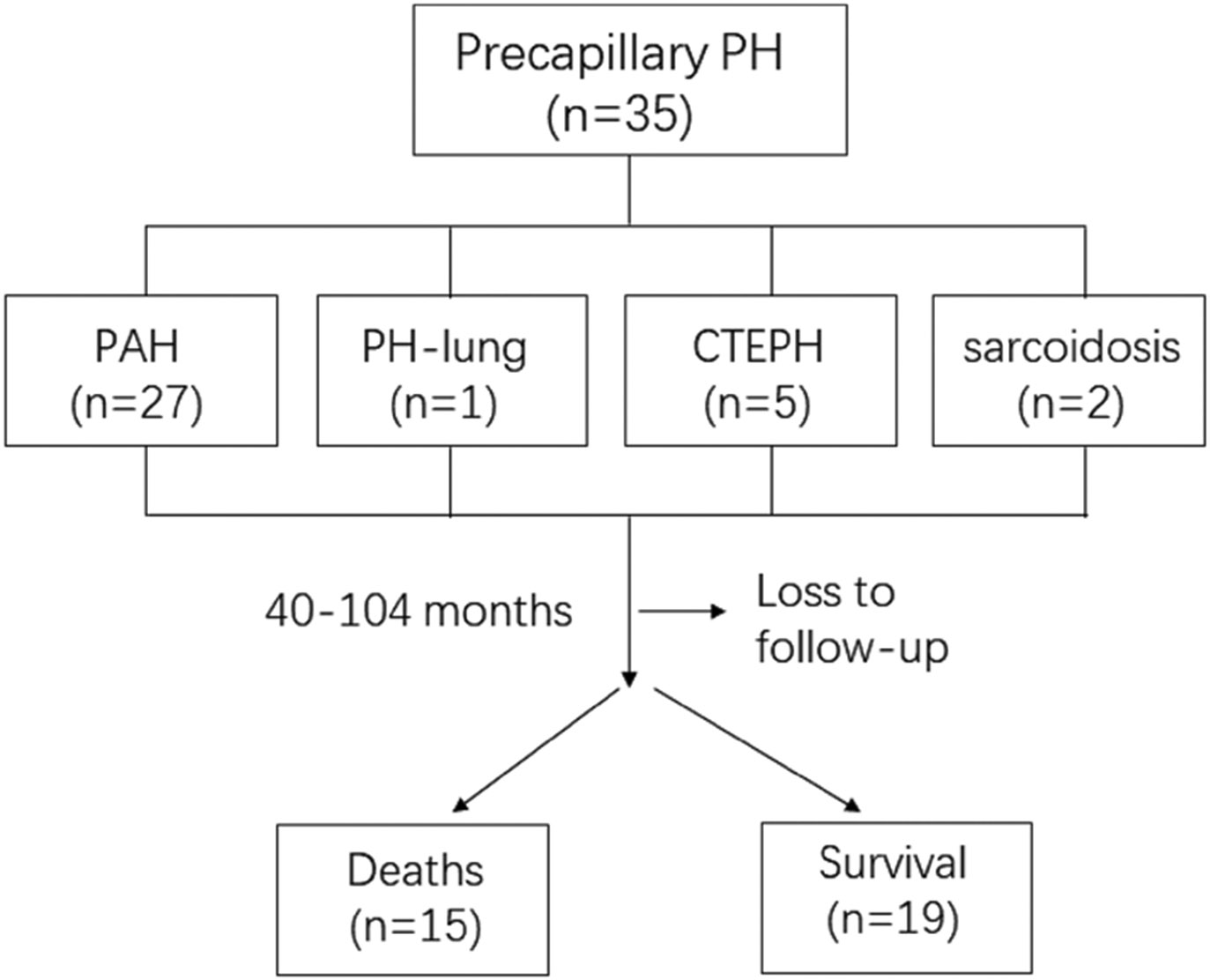
Patient flowchart. Thirty-five patients were enrolled, and native T1, T2, and T1ρ mapping using CMR was performed

A total of 35 patients were enrolled. The flowchart is shown in Fig. 1. The patients were grouped into: idiopathic PAH, *n* = 16; PAH associated with connective tissue disease, *n* = 10; simple congenital with unrepaired small atrial septal defect with persistent and out of proportion PAH, *n* = 1, patient with COPD, *n* = 1; chronic thromboembolic PH with nonsurgical/distal vessel disease, *n* = 5; PH with sarcoidosis (in the absence of LV involvement), *n* = 2. One patient was excluded due to loss to follow-up. The clinical characteristics and CMR functional parameters of the subjects are shown in Table 1. The median follow-up time was 79 (Interquartile range 40–103) months. During follow-up, 15 out of 34 patients died (cumulative event rate: 44.1%), of whom 5 died of heart failure, 4 died of sudden cardiac death, 3 died of pulmonary embolism, 2 died of pneumonia, and 1 died of lung cancer. RV functional parameters of PH patients are significantly different from healthy controls, including decreased RV ejection fraction (EF) (36.9 ± 12.8% vs. 54.7 ± 4.4%, *P* < 0.001), decreased RV/pulmonary artery (PA) coupling as evidenced by RV stroke volume (SV)/ RV end_systolic volume (ESV) (0.7 ± 0.4 vs. 1.2 ± 0.2, *P* < 0.001), and increased RV size evidenced by the ratio of RV end_diastolic volume (EDV) to LVEDV (1.9 ± 0.9 vs. 1.1 ± 0.1, *P* < 0.001). In addition, decreased LVEF (56.8 ± 8.5% vs. 61.6 ± 4.0%, *P* = 0.019), and decreased LV/aorta coupling evidence by LVSV/LVESV (1.4 ± 0.5 vs. 1.6 ± 0.3, *P* = 0.018) were also significant between the PH group and control group.

PAH: pulmonary arterial hypertension, PH-lung: PAH due to lung disease and/or hypoxia, CTEPH: chronic thromboembolic pulmonary hypertension.

### Native T1, T2, and T1ρ values are elevated in PH patients

**Fig. 2:**
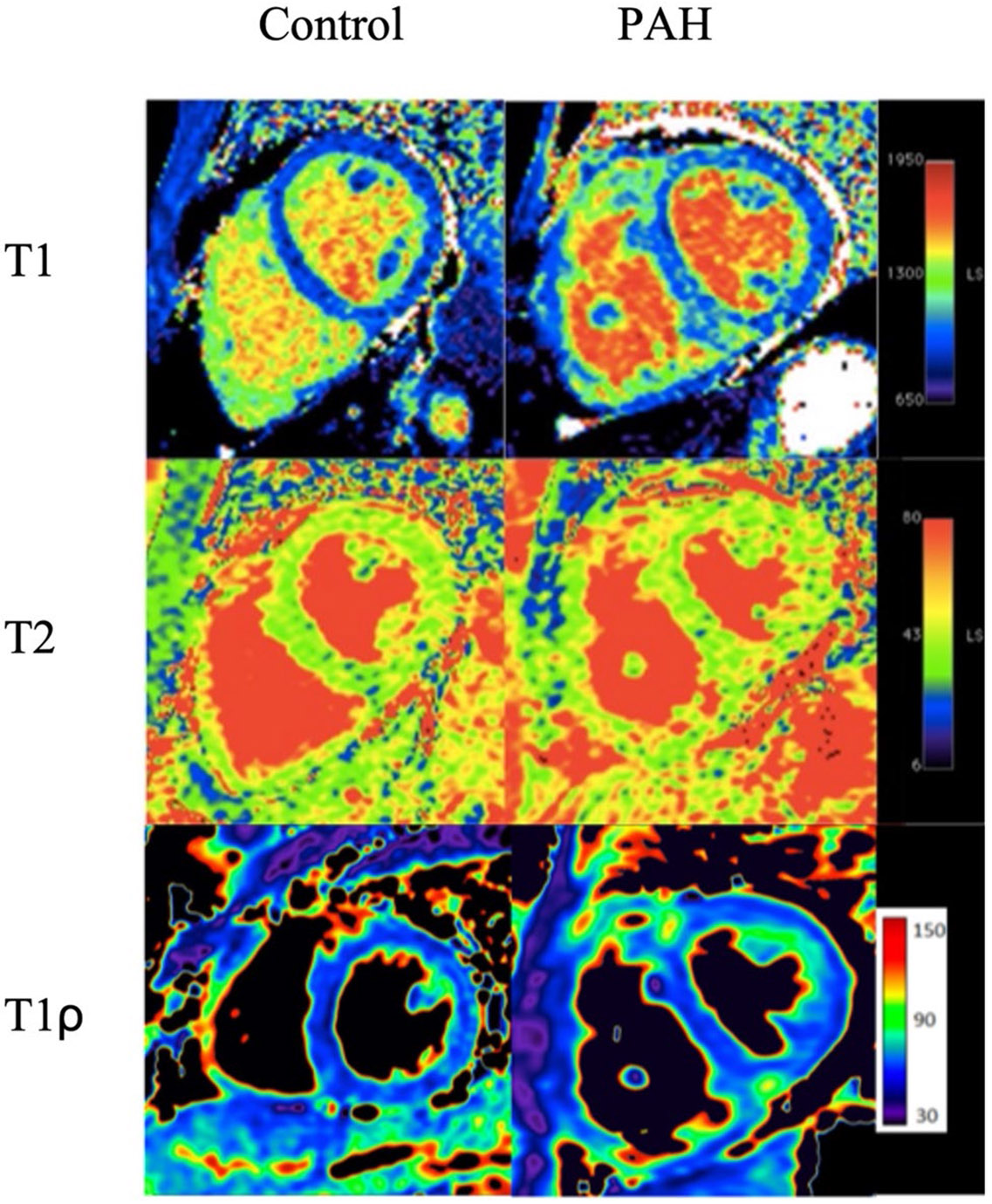
T1, T2, and T1ρ maps of control versus a patient with pulmonary arterial hypertension (PAH). Higher values seen as brighter areas are visualized in the right ventricular insertion points in PAH

Native T1, T2, and T1ρ values are shown in Table 2. As compared to controls, native T1, T2, and T1ρ values of all the ROIs were significantly higher, including RVIP, the septum, LV lateral wall, and RV free wall. For each ROIs, T1_RVIP, T2_RVIP, and T1ρ_RVIP bears the largest difference compared to controls. An example of the endogenous T1, T2, and T1ρ maps in a PH patient versus a control is shown in Fig. 2.

### Correlation between T1, T2, and T1ρ and CMR function parameters

The correlation between T1, T2, and T1ρ and CMR function parameters RVEDV indexed to body surface area (BSA), RVESV index, RVEF, RVSV/RVESV, and RVEDV/ LVEDV are shown in Table 3. T1, T2, and T1ρ at the RVIP showed the highest correlation with CMR functional parameters, compared with the values measured at septum, LV lateral wall, or RV free wall. The strongest positive correlation was found between T1ρ and RVESVi (*r* = 0.80, *P* < 0.001) and the strongest negative correlation was between T1ρ and RVEF (*r* = −0.80, *P* < 0.001).

### Time-dependent ROC analysis of the function parameters and tissue characteristic parameters for predicting all-cause mortality

The AUCs and the prediction thresholds of the LV and RV function parameters and tissue characteristic parameters (T1, T2, and T1ρ) for predicting the all-cause mortality se parameters were summarized in Table S1. Overall, the baseline T1_RVIP, T2_RVIP, and T1ρ_RVIP had good predictive power for all-cause mortality at different time points, and the AUCs of which were higher at most time points compared with LV and RV function parameters. Among the three tissue characteristic parameters, T1_RVIP had the highest accuracy in predicting mortality during the entire observation time (36–108 months). T2_RVIP of 56.1 ms showed the highest values indicating 3-year mortality with an AUC of 0.950 (sensitivity: 1.000, specificity 0.824). T1_RVIP of 1215.2 ms also showed high predicting power of 3-year mortality with AUC of 0.870 (sensitivity: 0.946, specificity 0.922) (Table S1).

### Univariate and multivariate Cox regression analysis of function parameters and mapping parameters

In univariate Cox regression analyses, all the three tissue relaxation parameters were significantly associated with all-cause mortality (T1_RVIP (per 5 ms): hazard ratio, 1.2, [1.0–1.3], *P* = 0.008; T2_RVIP (per 3 ms): hazard ratio, 2.1, [1.3–3.5], *P* = 0.004; T1ρ _RVIP (per 3 ms): hazard ratio, 1.2, [1.0–1.3], *P* = 0.008, respectively) (Table 4). In function parameters, only RVEDVi (per 20 ml/m^2^) showed significant relationship with outcome (hazard ratio, 1.2, [1.0–1.5], *P* = 0.049). NT-proBNP (per 100 pg/ml) also showed significant relationship with outcome (hazard ratio, 1.1, [1.0–1.2], *P* = 0.031).

We developed a multivariate Cox model to assess the independent prognostic significance of native T1_RVIP (5 ms), T2_RVIP (3 ms), and T1ρ_RVIP (3 ms), as well as RVEDVi (20 ml/m^2^) and NT-proBNP (100 pg/ml) (Table 5). T2_RVIP remained as the only independent predictor of allcause mortality (T2 (3 ms): hazard ratio, 3.0 [1.2–7.6], *P* = 0.018) (Table 5).

We next developed a multivariate model including RVEDVi and each one of the tissue characteristic parameters. T1_RVIP (5 ms), T2_RVIP (3 ms) and T1ρ_RVIP (3 ms) were respectively significant predictor for mortality after adjusting for RVEDVi (20 ml/m^2^) (*P* = 0.030 for T1_RVIP (5 ms), *P* = 0.011 for T2_RVIP (3 ms) and *P* = 0.031 for T1ρ_RVIP (3 ms)). RVEDVi (20 ml/m^2^) didn’t show significant predictive value for mortality (Table 5).

We then included NT-proBNP (100 pg/ml) and each one of the tissue characteristic parameters into a multivariate model. T2_RVIP (3 ms) remained an independent predictor for mortality after adjusting for NT-proBNP (100 pg/ml) (hazard ratio, 2.5, [1.1–5.9], *P* = 0.035) while T1_RVIP (5 ms) and T1ρ_RVIP (3 ms) showed no predictive value adjusted by NT-proBNP (Table 5).

### Kaplan-Meier survival analyses of LV and RV functional parameters

**Fig. 3:**
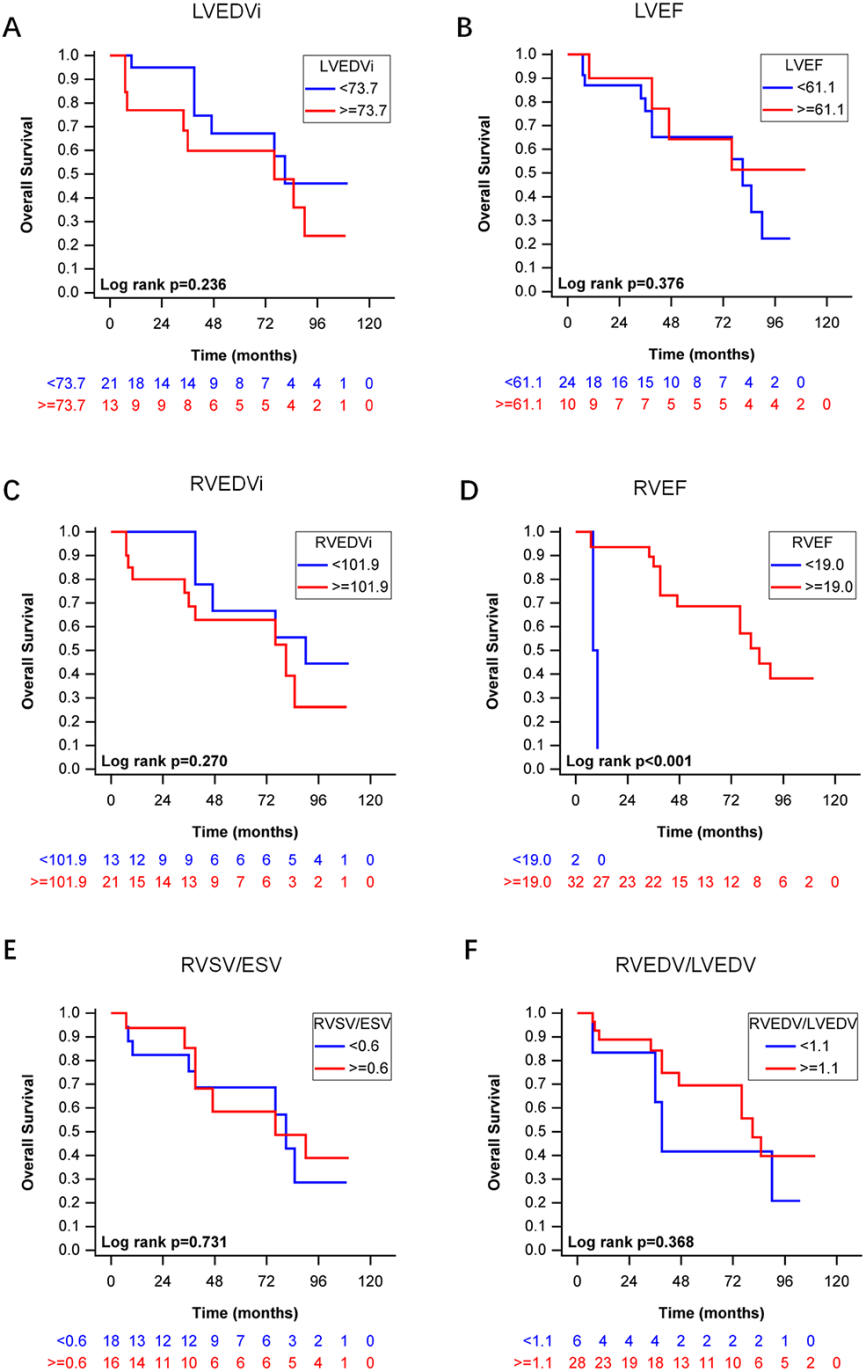
Kaplan-Meier curves analysis for all-cause mortality using LV and RV functional parameters

According to time-dependent ROC curves, the cutoff values of LV and RV functional parameters with the highest AUC were selected in Kaplan-Meier survival analyses. All-cause mortality was significantly lower in patients with RVEF ≥ 19.0% (*P* < 0.001) compared with RVEF < 19.0% (Fig. 3). Other CMR function parameters were not significantly associated with all-cause mortality.

### Kaplan-Meier survival analyses of T1, T2, and T1ρ

**Fig. 4:**
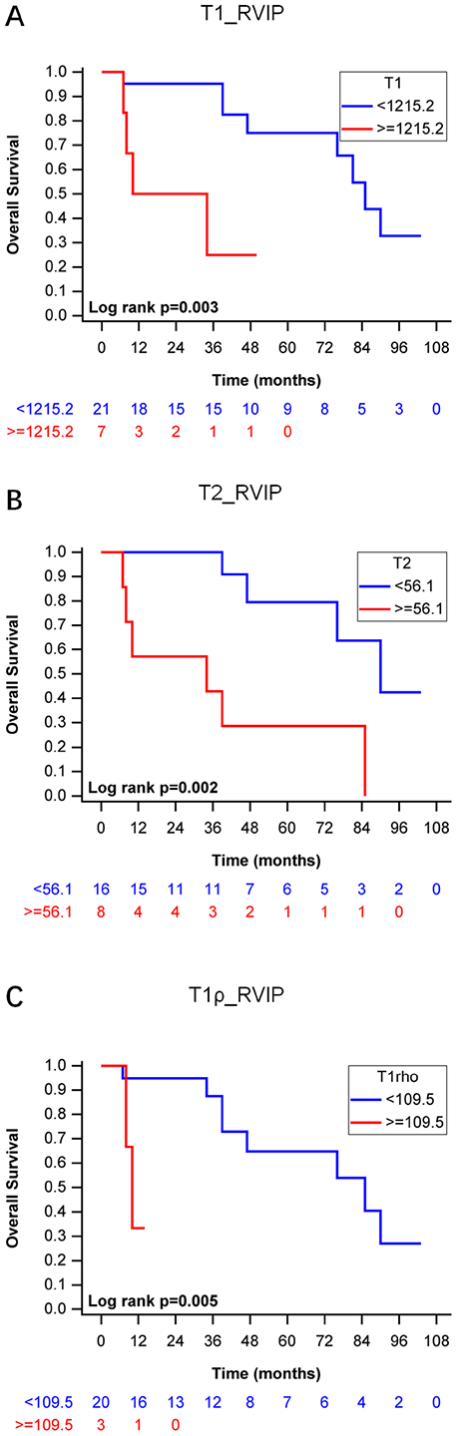
Kaplan-Meier curves for predicting overall survival using native T1, T2, and T1ρ values at RVIP. (A-C). Native T1 values < 1215.2 ms predicts high incidence of overall survival (A). Native T2 values < 56.1 ms predicts high incidence of overall survival (B). Native T1ρ values < 109.5 ms predicts high incidence of overall survival (C)

According to time-dependent ROC curves, the cutoff values of T1, T2, and T1ρ with the highest AUC were selected as cutoffs in the Kaplan-Meier survival analyses. All the three tissue relaxation parameters (T1_RVIP, T2_RVIP, and T1ρ_RVIP) could clearly differentiate all-cause mortality of PH patients respectively. Patients with native T1_RVIP < 1215.2 ms (*P* = 0.003), native T2_RVIP < 56.1 ms (*P* = 0.002), and T1ρ_RVIP < 109.5 ms (*P* = 0.005) showed a better survival than those with native T1_RVIP ≥ 1215.2 ms, native T2_RVIP ≥ 56.1 ms, and T1ρ_RVIP ≥ 109.5 ms, respectively (Fig. 4).

### Reproducibility

Overall, the intra- and inter-observer reproducibility results were good to excellent as shown in Table S2. The intraclass correlation coefficients for the T1_RVIP, T2_RVIP, and T1ρ_RVIP were: 0.983, 0.968 and 0.985, respectively. The interclass correlation coefficients for the T1_RVIP, T2_RVIP, and T1ρ_RVIP were: 0.925, 0.903, and 0.977, respectively.

## Discussion

Our study employed native T1, T2, and T1ρ mapping to evaluate the cardiac tissue characterization and their prognostic values during a long-term follow-up in precapillary PH. We demonstrated that the severity of RV dysfunction in PH patients and the outcomes of these patients are correlated with native tissue mapping parameters and T2 RVIP is an independent predictor of all-cause mortality in this small study.

Compared to controls, there were significant elevations in T1, T2, and T1ρ values in all the ROIs including the RVIPs, septum, RV free wall, and LV free wall. Strong correlations were found among T1_RVIP, T2_RVIP, and T1ρ_RVIP and functional parameters RVEDVi, RVESVi, RVEF, RVSV/ESV, and RVEDV/LVEDV. More importantly, increased T1_RVIP, T2_RVIP, and T1ρ_RVIP are significant predictors for all-cause mortality in precapillary PH patients. T2_RVIP of 56.1 ms showed the highest values indicating 3-year mortality with an AUC of 0.950 (sensitivity: 1.000, specificity 0.824) and T1_RVIP of 1215.2 ms had an AUC of 0.870 (sensitivity: 0.946, specificity 0.922) for all-cause mortality. T2_RVIP is independently predictive of all-cause mortality in addition to NT-proBNP and RVEDVi during long-term follow-up. These tissue characterization parameters may provide additional important information to clinical assessment, risk stratification, and prognosis for precapillary PH patients.

In this study, we utilized three CMR native mapping indices (T1, T2, and T1ρ) to comprehensively evaluate the cardiac tissue characterization, and explore their prognostic values in PH. Previous studies have shown increased T1 and T2 values at RVIP and correlated with cardiac function and hemodynamics in PAH patients [14-16] but only a few reports focused on the prognostic value of T1 and T2. There has been no report on the prognostic potential of T1ρ in this population. In a recent study including 5 clusters of systemic sclerosis patients, native myocardial T1 at the interventricular septum was an independent predictor of mortality after adjustment for age and the presence of PH [21]. In a study which included many different types of PH, RVIP was the most successful T1 region for discriminating PH patients from healthy subjects, but did not contribute to overall mortality prediction [22]. Native T1 and T2 independently predicted the combined endpoint of event occurrence/recurrence or cardiovascular mortality in high-risk systemic sclerosis [23]. T1ρ has been recently used as a biomarker to quantify acute and chronic myocardial injuries of ischemic or non-ischemic origins in our and other studies [17, 24, 25]. In the present study, we found elevated T1, T2 and T1ρ values of multiple regions in both LV and RV (RVIP, septum, LV lateral wall and RV free wall), which all correlated significantly with RV dysfunction and RV remodeling.

Elevated T1, T2, and T1ρ values at multiple regions of both LV and RV are results of aggregated mechanical stress, tissue deformation, and pathogenesis progression. RV pressure overload exert a pronounced mechanical and structural changes to the LV and RV myocardium in both animal and human studies [26-28]. Consistent stress in turn leads to myocardial injury, impaired extracellular matrix, myofibroblast activation and fibrosis. RVIP bears the most mechanical stress from LV and RV, which derived from combination of progressive RV dilation, LV deformation and paradoxical septum motion. Elevated T1, T2, and T1ρ values at multiple regions of both LV and RV are representative of extensive impairment and diffuse histological changes such as fibrosis and inflammation. T1, T2, and T1ρ value at RVIP are more pronounced with RV dysfunction, compared to the other regions of LV and RV. Myocardial disarray and plexiform fibrosis at RVIP were shown in autopsies of patients with dilated cardiomyopathy and in an experimental PH porcine model [29-31]. The histological changes resulted increased T1_RVIP on CMR, which correlated with collagen volume at RVIP [30]. Utilizing the distinct non-overlapping but non-orthogonal physical properties of the myocardial T1, T2, and T1ρ values in characterizing myocardial tissue, our multiparametric approach studied the complex underlying pathophysiology by assessing diffuse interstitial fibrosis, myocardium edema, and macromolecular changes in PH. The potential of elevated T1, T2, and T1ρ at RVIP as prognostic markers of outcomes are highly relevant.

Our study has several limitations. First, this was a singlecenter prospective study with a small number of patients. The small study size limited more variables to be included in the multivariable Cox regression analysis and sub-group analysis. Second, the average age of the control group was younger than PH group. However, no significant association between age and native T1 or T2 were identified [32, 33]. Only a weak (*r* = 0.27) but significant association with age was identified in T1ρ in a Chinese population [34]. Further studies will be needed to clarify the age dependency of tissue mapping parameters, but the magnitude of age dependency is unlikely to change the conclusion of this study due to high elevation of these values in the patient population. Third, hemodynamic parameters from right heart catheterization were not included because concurrent catheterization was not performed. These parameters would have strengthened the physiological interpretation and analysis. Fourth, we did not use gadolinium in this study to limit scan time and improve tolerability for patients, thus, we could not directly compare the native tissue mapping with LGE for the prognosis of PH. The prognostic significance of native T1, T2, and T1ρ values at RVIPs needs further validation.

## Conclusion

During long-term follow-up, non-contrast tissue relaxation parameters T1, T2, and T1ρ at the RVIP are found to be highly correlated with RV functional parameters in pre-capillary PH patients. T2_RVIP is shown in this small study to be independently associated with long-term outcome and may be a novel prognostic parameter for precapillary PH. However, this will need to be validated in larger studies.

## Supplementary Material

Supplemental

## Figures and Tables

**Table 1:** Baseline patient characteristics

Characteristics	Patient (35)	Control (15)	*p*-Value
Age	58.0 ± 13.4	43.5 ± 13.0	0.002
Male (%)	12 (34)	7 (47)	0.413
BSA (m^2^)	2.0 ± 0.3	1.9 ± 0.3	0.832
NT-proBNP (pg/ml)	1093.7 ± 1193.1	/	/
6MWD (m)	339.2 ± 128. 8	/	/
Type of PAH		/	/
Idiopathic PAH	16(46)	/	/
PAH associated with CTD	10(29)	/	/
CTEPH-nonsurgical/distal vessel disease	5(14)	/	/
Simple congenital such as ASD/VSD	1(3)	/	/
Group 3 patients	1(3)	/	/
Group 5 PH sarcoidosis	2(6)	/	/
WHO Grade		/	/
II	19(54)	/	/
III	13(37)	/	/
IV	3(9)	/	/
Cardiac Function			
LVEDV (ml)	142.7 ± 36.4	160.1 ± 38.4	0.125
LVESV (ml)	64.4 ± 26.9	61.5 ± 15.7	0.992
LVEDVi (ml/m^2^)	73.2 ± 16.0	82.0 ± 13.1	0.045
LVESVi (ml/m^2^)	33.8 ± 11.9	31.5 ± 5.5	0.992
LVEF (%)	56.8 ± 8.5	61.6 ± 4.0	0.019
LVSV/LVESV	1.4 ± 0.5	1.6 ± 0.3	0.018
RVEDV (ml)	254.0 ± 104.7	178.7 ± 46.5	0.010
RVESV (ml)	170.9 ± 101.4	80.9 ± 21.4	0.001
RVSV (ml)	83.2 ± 16.5	97.7 ± 27.6	0.058
RVEDVi (ml/m^2^)	132.5 ± 61.1	91.3 ± 17.3	0.011
RVESVi (ml/m^2^)	89.0 ± 56.3	41.4 ± 8.5	0.001
RVEF (%)	36.9 ± 12.8	54.7 ± 4.4	< 0.001
RVSV/RVESV	0.7 ± 0.4	1.2 ± 0.2	< 0.001
RVEDV/LVEDV	1.9 ± 0.9	1.1 ± 0.1	< 0.001

CMR: cardiovascular magnetic resonance imaging, BSA: body surface area, 6MWD: 6-minute walking distance, PAH: pulmonary artery hypertension, LV: left ventricle, RV: right ventricle, EDVi: end diastolic volume index, ESVi: end systolic volume index, SV: stroke volume, EF: ejection fraction

**Table 2:** Comparison of regional values of nativeT1, T2 and T1ρ to controls

		Patients	Control	*p*-Value
T1 (ms)	Average RVIP	1176.9 ± 85.6	1006.5 ± 54.4	< 0.001
	Septum	1079.0 ± 63.9	1005.2 ± 22.8	< 0.001
	LV Lateral wall	1037.3 ± 43.4	984.6 ± 25.1	< 0.001
	RV Free wall	1065.8 ± 35.9	977.4 ± 65.0	< 0.001
T2 (ms)	Average RVIP	55.7 ± 5.7	44.7 ± 3.1	< 0.001
	Septum	48.2 ± 3.7	44.5 ± 2.0	0.002
	LV Lateral wall	49.2 ± 4.2	44.0 ± 2.3	< 0.001
	RV Free wall	48.8 ± 4.1	44.8 ± 4.0	0.009
T1ρ (ms)	Average RVIP	97.6 ± 21.6	66.9 ± 7.8	< 0.001
	Septum	78.6 ± 16.0	62.8 ± 7.3	< 0.001
	LV Lateral wall	77.0 ± 14.3	62.6 ± 6.3	< 0.001
	RV Free wall	81.9 ± 14.5	67.9 ± 7.6	0.005

RVIP: right ventricular insertion point. LV: left ventricle. RV: right ventricle

**Table 3:** Correlations between T1, T2, T1ρ and cardiac functional parameters

		RVEDVi		RVESVi		RVEF		RVSV		RVSV/RVESV		RVEDV/LVEDV	
		r	*p*	r	*p*	r	*p*	*r*	*p*	r	*p*	r	*p*
T1	Aver RVIP	0.76	< 0.001	0.76	< 0.001	−0.67	< 0.001	0.13	0.506	−0.67	< 0.001	0.70	< 0.001
	Septum	0.64	< 0.001	0.61	< 0.001	−0.51	0.005	0.14	0.482	−0.51	0.004	0.46	0.012
	LV Lateral	0.45	0.015	0.42	0.025	−0.29	0.122	0.13	0.490	−0.30	0.120	0.25	0.193
	RV Wall	0.64	< 0.001	0.68	< 0.001	−0.66	< 0.001	0.12	0.532	−0.66	< 0.001	0.58	0.001
T2	Aver RVIP	0.59	0.002	0.58	0.002	−0.53	0.007	0.08	0.70	−0.53	0.006	0.60	0.002
	Septum	0.26	0.174	0.26	0.176	−0.20	0.302	0.12	0.551	−0.19	0.324	0.38	0.044
	LV Lateral	0.22	0.249	0.18	0.353	−0.04	0.847	0.20	0.296	−0.04	0.848	0.23	0.228
	RV Wall	0.16	0.404	0.16	0.408	−0.12	0.549	0.22	0.246	−0.11	0.564	0.32	0.096
Tiρ	Aver RVIP	0.73	< 0.001	0.80	< 0.001	−0.80	< 0.001	0.10	0.654	−0.78	< 0.001	0.78	< 0.001
	Septum	0.48	0.008	0.47	0.010	−0.37	0.049	0.21	0.270	−0.37	0.048	0.59	0.001
	LV Lateral	0.42	0.025	0.38	0.044	−0.25	0.194	0.33	0.079	−0.25	0.188	0.47	0.011
	RV Wall	0.45	0.015	0.44	0.017	−0.37	0.046	0.17	0.386	−0.37	0.049	0.55	0.002

RVIP: right ventricle insertion point, LV: left ventricle, RV: right ventricle, EDVi: end diastolic volume index, ESVi: end systolic volume index, SV: stroke volume, EF: ejection fraction

**Table 4:** Univariable Cox regression of clinical and imaging parameters for predicting all-cause mortality

Variables	Univariable Analysis	
	Unadjusted HR (95%CI)	*p*-Value
Age (years)	1.0 (1.0–1.0)	0.922
Male (%)	1.1 (0.3–4.3)	0.899
BSA (m^2^)	0.3 (0.0–3.8)	0.372
NT-proBNP (per 100 pg/ml)	1.1 (1.0–1.2)	**0.031**
6MWD	1.0 (1.0–1.0)	0.700
Type of PAH	0.5 (0.1–2.1)	0.354
WHO Class	2.7 (0.9–7.9)	0.073
LVEDVi	1.0 (1.0–1.1)	0.674
LVESVi	1.0 (1.0–1.1)	0.600
LVEF(%)	1.0 (0.9–1.1)	0.683
LVSV/LVESV	0.6 (0.1–2.6)	0.481
RVEDVi (per 20 ml/m^2^)	1.2 (1.0–1.5)	**0.049**
RVEF (%)	1.0 (0.9–1.0)	0.114
RVSV	1.0(0.9–1.0)	0.824
RVSV/RVESV	0.2 (0.0–1.5)	0.112
RVEDV/LVEDV	2.2 (0.9–5.1)	0.067
T1 (per 5 ms)	1.2 (1.0–1.3)	**0.008**
T2 (per 3 ms)	2.1 (1.3–3.5)	**0.004**
T1p (per 3 ms)	1.2 (1.0–1.3)	**0.008**

HR: hazard ratio, other abbreviations as in Table 1

**Table 5:** Multivariate Cox regression of clinical and imaging parameters for predicting all-cause mortality

	Adjusted HR (95%CI)	*p*-Value	Adjusted HR (95%CI)	*p*-Value	Adjusted HR (95%CI)	*p*-Value	Adjusted HR (95%CI)	*p*-Value	Adjusted HR (95%CI)	*p*-Value	Adjusted HR (95%CI)	*p*-Value	Adjusted HR (95%CI)	*p*-Value	Adjusted HR (95%CI)	*p*-Value	Adjusted HR (95%CI)	*p*-Value	Adjusted HR (95%CI)	*p*-Value
NT-proBNP Every 100pg/ml	1.0 (0.8–1.1)	0.491	1.0 (0.9–1.1)	0.751	1.008 (0.882–1.151)	0.910	1.0 (0.9–1.2)	0.692							1.0 (0.9–1.1)	0.927	1.0 (0.9–1.1)	0.626	1.0 (0.9–1.1)	0.857
RVEDVi Every 20 ml/m2	0.6 (0.4–1.0)	0.059	0.9 (0.6–1.3)	0.670	0.813 (0.544–1.215)	0.313	0.9 (0.6–1.2)	0.457	1.0 (0.7–1.3)	0.765	0.8 (0.6–1.2)	0.275	0.9 (0.7–1.2)	0.526						
T1_RVIP Every 5 ms	1.1 (0.9–1.3)	0.593	1.2 (1.0–1.4)	0.056					1.2 (1.0–1.4)	**0.030**					1.2 (1.0–1.4)	0.064				
T2_RVIP Every 3 ms	3.1 (1.0–10.2)	0.058			3.030 (1.205–7.621)	**0.018**					3.1 (1.3–7.4)	**0.011**					2.5 (1.1–5.9)	**0.035**		
T1ρ_RVIP Every 3 ms	1.2 (1.0–1.6)	0.097					1.2 (1.0–1.4)	0.073					1.2 (1.0–1.4)	**0.031**					1. 1 (1.0–1.3)	0.092

## Data Availability

The data that support the findings of this study are not openly available due to reasons of sensitivity and are available from the corresponding author upon reasonable request.

## Abbreviations

6MWD: 6-minute walking distance
BSA: body surface area
CMR: cardiovascular magnetic resonance imaging
EDVi: end diastolic volume index
EF: ejection fraction
ESVi: end systolic volume index
HRs: hazards ratios
ICC: intra class correlation coefficient
LGE: late gadolinium enhancement
LV: left ventricle
MOLLI: modified Look-Locker inversion
PAH: pulmonary artery hypertension
PVR: pulmonary vascular resistance
ROC: receiver operating characteristic
RV: right ventricle
RVIP: right ventricle insertion point
SV: stroke volume
