# Supplementary material for: Long-term prognostic value of native myocardial tissue relaxation parameters (T1, T2, and T1ρ) in patients with precapillary pulmonary hypertension
Source: Int J Cardiovasc Imaging. Author manuscript; Available in PMC 2025 Aug 13. (PMC12346376; doi:10.1007/s10554-025-03451-5)
Supplement: Supplemental [file NIHMS2095360-supplement-Supplemental.docx]

Supplemental material:

Figure S1. Examples of region of interest (ROI) for a control and a PH patient. (A). The ROIs of a health control. (B). The ROIs of a PH patient. Red: upper RVIP; yellow: lower RVIP; green: septum; blue: LV lateral wall; purple: RV free wall.


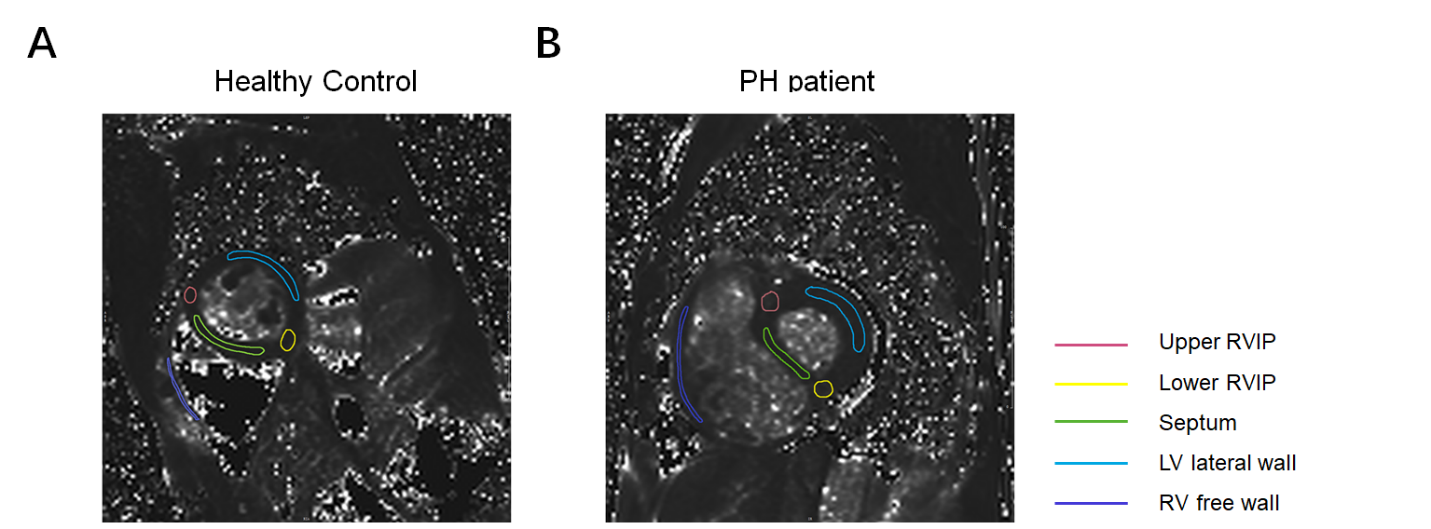


Table S1. The AUC, sensitivity, and specificity for the best threshold of function parameters and tissue characteristic parameters.

|  | **AUC (best threshold)** | **Sensitivity** | **Specificity** |
| --- | --- | --- | --- |
| LVEDVi | 0.72 (73.7) | 0.777 | 0.715 |
| LVESVi | 0.71 (35.9) | 0.695 | 0.805 |
| LVEF | 0.73 (61.1) | 0.916 | 0.588 |
| LVSV/ESV | 0.62 (1.8) | 1.000 | 0.422 |
| RVEDVi | 0.64 (101.9) | 0.982 | 0.472 |
| RVESVi | 0.61 (49.0) | 0.972 | 0.396 |
| RVEF | 0.60 (19.0) | 0.298 | 1.000 |
| RVSV/ESV | 0.62 (0.60) | 0.812 | 0.503 |
| RVEDV/LVEDV | 0.54 (1.10) | 0.372 | 0.945 |
| T1_RVIP | 0.87 (1215.2) | 0.946 | 0.922 |
| T2_RVIP | 0.95 (56.1) | 1.000 | 0.824 |
| T1ρ_RVIP | 0.66 (109.5) | 0.429 | 0.945 |

Table S2. Intra-observer and inter-observer reproducibility of CMR parameters.

|  |  |  | ICC | Lower Bound | Upper Bound | p |
| --- | --- | --- | --- | --- | --- | --- |
| Intra | T1 | Average RVIP | 0.983 | 0.935 | 0.996 | <0.001 |
|  |  | septum | 0.960 | 0.850 | 0.990 | <0.001 |
|  |  | LV lateral wall | 0.953 | 0.822 | 0.988 | <0.001 |
|  |  | RV free wall | 0.829 | 0.454 | 0.955 | 0.001 |
|  | T2 | Average RVIP | 0.968 | 0.876 | 0.992 | <0.001 |
|  |  | septum | 0.870 | 0.563 | 0.966 | <0.001 |
|  |  | LV lateral wall | 0.820 | 0.432 | 0.952 | 0.001 |
|  |  | RV free wall | 0.751 | 0.270 | 0.930 | 0.004 |
|  | T1ρ | Average RVIP | 0.985 | 0.941 | 0.996 | <0.001 |
|  |  | septum | 0.939 | 0.777 | 0.985 | <0.001 |
|  |  | LV lateral wall | 0.951 | 0.817 | 0.988 | <0.001 |
|  |  | RV free wall | 0.972 | 0.893 | 0.993 | <0.001 |
| Inter | T1 | Average RVIP | 0.925 | 0.729 | 0.981 | <0.001 |
|  |  | septum | 0.886 | 0.609 | 0.970 | <0.001 |
|  |  | LV lateral wall | 0.884 | 0.602 | 0.970 | <0.001 |
|  |  | RV free wall | 0.668 | 0.110 | 0.906 | 0.012 |
|  | T2 | Average RVIP | 0.903 | 0.659 | 0.975 | <0.001 |
|  |  | septum | 0.915 | 0.697 | 0.978 | <0.001 |
|  |  | LV lateral wall | 0.788 | 0.353 | 0.943 | 0.002 |
|  |  | RV free wall | 0.663 | 0.102 | 0.904 | 0.013 |
|  | T1ρ | Average RVIP | 0.977 | 0.910 | 0.994 | <0.001 |
|  |  | septum | 0.824 | 0.442 | 0.953 | 0.001 |
|  |  | LV lateral wall | 0.903 | 0.660 | 0.975 | <0.001 |
|  |  | RV free wall | 0.928 | 0.738 | 0.982 | <0.001 |

RVIP: right ventricle insertion point, LV: left ventricle, RV: right ventricle.
